# Supplementary material for: Host genetic regulation of human gut microbial structural variation
Source: Nature. 2024 Jan 3;625(7996):813–21. doi: 10.1038/s41586-023-06893-w (PMC10808065; doi:10.1038/s41586-023-06893-w)
Supplement: Supplementary file 1 — Supplementary Notes 1–4 and Figs. 1 and 2. [file 41586_2023_6893_MOESM1_ESM.pdf]

nature portfolio

https://doi.org/10.1038/s41586-023-06893-w

Supplementary information

Host genetic regulation of human gut microbial structural variation

In the format provided by the authors and unedited

Supplementary Information

Host Genetic Regulation of Human Gut Microbial Structural Variation

Authors

Daria V. Zhernakova[1,*], Daoming Wang[1,2,*], Lei Liu[3,*], Sergio Andreu-Sánchez[1,2], Yue Zhang[1,2], Angel J. Ruiz-Moreno[1,2], Haoran Peng[1], Niels Plomp[3,4], Ángela Del Castillo-Izquierdo[1,3], Ranko Gacesa[1,4], Esteban A. Lopera-Maya[1], Godfrey S. Temba[5,6,7], Vesla I. Kullaya[6,8], Sander S. van Leeuwen[9], Lifelines Cohort Study, Human Functional Genomics Project, Ramnik J. Xavier[10], Quirijn de Mast[5,7], Leo A.B. Joosten[5,11], Niels P. Riksen[5], Joost H.W. Rutten[5], Mihai G. Netea[5,7,12,13], Serena Sanna[1,14], Cisca Wijmenga[1], Rinse K. Weersma[4], Alexandra Zhernakova[1], Hermie J.M. Harmsen[3,#], and Jingyuan Fu[1,2,#,$]

Affiliations

[1] Department of Genetics, University of Groningen, University Medical Center Groningen, Groningen 9713 AV, the Netherlands

[2] Department of Pediatrics, University of Groningen, University Medical Center Groningen, Groningen 9713 AV, the Netherlands

[3] Department of Medical Microbiology and Infection Prevention, University of Groningen, University Medical Center Groningen, Groningen 9713 GZ, the Netherlands

[4] Department of Gastroenterology and Hepatology, University of Groningen, University Medical Center Groningen, Groningen 9713 GZ, the Netherlands

[5] Department of Internal Medicine, Radboud University Medical Center, Nijmegen 6500 HB, the Netherlands

[6] Department of Medical Biochemistry and Molecular Biology, Kilimanjaro Christian Medical University College, P.O. Box 2240, Moshi, Tanzania

[7] Radboud Center for Infectious Diseases, Radboud University Medical Center, Nijmegen 6500 HB, the Netherlands

[8] Kilimanjaro Clinical Research Institute, Kilimanjaro Christian Medical Center, Moshi, Tanzania

[9] Department of Laboratory Medicine, University of Groningen, University Medical Center Groningen, Groningen 9713 AV, the Netherlands

[10] Broad Institute of MIT and Harvard, Cambridge, MA 02142, USA; Center for Computational and Integrative Biology, Department of Molecular Biology, Massachusetts General Hospital, Boston, MA 02114, USA

[11] Department of Medical Genetics, Iuliu Haţieganu University of Medicine and Pharmacy, Cluj-Napoca 400000, Romania

[12] Department of Immunology and Metabolism, Life and Medical Sciences Institute, University of Bonn, Bonn 53113, Germany

[13] Human Genomics Laboratory, Craiova University of Medicine and Pharmacy, Craiova 200349, Romania

[14] Institute for Genetic and Biomedical Research, National Research Council, Cagliari, Italy

* These authors contributed equally

# Shared senior authorship

$ Lead Contact

Table of contents

Supplementary Notes 3

Supplementary Note 1. Comparison of SV heritability with species abundance heritability 3

Supplementary Note 2: Replication of *ABO*–*F. prausnitzii* associations in a Tanzanian cohort 3

Supplementary Note 3: Key genes in the GalNAc utilization pathway 3

Supplementary Note 4. Transmission of the GalNAc SV 4

Supplementary Figures 5

Supplementary Figure 1: Graphical representation of the aligned gene regions with SVs of 12 *F. prausnitzii* strains 5

Supplementary Figure 2: Prediction of mobilizable genomic islands in the GalNAc region of *F. prausnitzii* 6

Supplementary Notes

Supplementary Note 1. Comparison of SV heritability with species abundance heritability

Here, we compared SV heritability with the heritability of the abundances of the corresponding species previously estimated for the same cohort[8]. Note that we corrected for species abundance in the heritability estimation of SVs, so the estimated SV heritability is independent of the host genetic effect on species abundance. For the heritable SVs, heritability of the abundance of the corresponding species seems to be either lower or not significant (**Extended Data Fig. 2**), suggesting there is an additional effect of host genetics on microbial SV level. For instance, for the most heritable dSV of *F. prausnitzii* (h^2 = 0.38, P_{DMP} = 9.55x10^{-6}), the previously reported heritability of *F. prausnitzii* abundance was only 0.13 (P_{DMP} = 0.02)[8]. For some species that are heritable on the abundance level we also detected nominally significant SV heritability even after correcting for species abundance. For example, for *Akkermansia muciniphila*, with previously reported abundance heritability h^2 = 0.3, we found several vSVs showing nominally significant heritability ranging between 0.25 and 0.56 (**Extended Data Fig. 2** and **Supplementary Table 5**).

Supplementary Note 2: Replication of *ABO–F. prausnitzii* associations in a Tanzanian cohort

The genetic associations of *F. prausnitzii* SVs were replicated in the 300 TZFG cohort. SVs of *F. prausnitzii* were detectable in 201 Tanzanian individuals at both similar and different frequencies to those seen in the Dutch cohorts (**Extended Data Fig. 3b**), and we detected 156 associations with the *ABO* locus at a nominally significant level (P < 0.05; **Supplementary Table 8**). The top-associated SV of *F. prausnitzii* in 300TZFG was dSV 575–577, and its association with rs550057 was significant in both the Dutch meta-analysis (P = 2.44x10^{-23}) and 300TZFG (P = 9.11x10^{-3}) (**Extended Data Fig. 3c**). The top association of the *F. prausnitzii* 577–579 dSV in the Dutch cohort with rs635634 was not significant in Tanzanian samples (P = 0.12). However, this deletion was nominally associated with other SNPs in both the Dutch and Tanzanian samples, for example rs1633513 ($P_{300TZFG}$ = 0.039, P_{Dutch_meta} = 1.59x10^{-4}) (**Extended Data Fig. 3d**), and these two SNPs are not in linkage disequilibrium (LD) in European and African populations (LD r^2_{AFR} = 0.04, r^2_{EUR} = 0.06) but may differentially represent blood types in the two populations.

Supplementary Note 3: Key genes in the GalNAc utilization pathway

The GalNAc utilization pathway can be divided into the initial cleavage from A-antigen (step 0) and the five key steps of GalNAc utilization (steps 1–5). In **Step 0**, the gene *GH109* encodes an α-N-acetylgalactosaminidase, which carries out hydrolysis for the cleavage of A-antigen and the release of GalNAc. In **Step 1. GalNAc uptake and trans-membrane transport**, four genes – *agaF*, *agaV*, *agaC* and *agaD* – are

homologous to the four necessary subunits of the GalNAc PTS system II complex protein (Enzyme II^{aga}). Enzyme II^{aga} takes up exogenous GalNAc and releases the phosphate ester N-acetyl-D-galactosamine 6-phosphate (GalNAc6P) into the cell cytoplasm in preparation for metabolism. In **Step 2. Hydrolysis of GalNAc6P**, the *nagA* gene encodes N-acetyl-glucosamine-6-phosphate deacetylase, which catalyzes the hydrolysis of the N-acetyl group of GalNAc6P to yield D-galactosamine 6-phosphate (GalN6P) and acetate. In **Step 3. Isomerization-deamination of GalN6P**, the gene *agaS* encodes the D-galactosamine-6-phosphate deaminase that catalyzes the isomerization-deamination of GalN6P to form D-tagatose 6-phosphate (T6P) and ammonium ion. In **Step 4. Phosphorylation of T6P**, the gene *lacC* (also known as *pfkB* or *fruK*) encodes the T6P kinase that responds to the formation of tagatose 1,6-bisphosphate (TBP) from T6P with energy exchange. This gene is also involved in the catalytic activity from fructose 6-phosphate (F6P) to fructose-1,6-bisphosphate (FBP). In **Step 5. Synthesis of final products**, the catalytic subunits of the tagatose-1,6-biphosphate aldolase (TBPA), *gatY-kbaY* or *gatZ-kbaZ*, can synthesize D-glyceraldehyde 3-phosphate (DHAP) and glycerone phosphate (GAP) from TBP.

Supplementary Note 4. Transmission of the GalNAc SV

We wondered whether the GalNAc-containing SV region could be transmitted between bacteria or between hosts. We first noticed that the SV region is not lineage-specific and can be seen in all major phylogenetic branches of *F. prausnitzii* in a tree constructed based on conservative marker genes (see **Methods**) (**Fig. 3c**). This was further confirmed by a clear discrepancy between the phylogenetic distances of GalNAc utilization genes compared to those of species marker genes (**Extended Data Fig. 6a**; phylogenetic distance = 0.73): the divergence of the GalNAc utilization genes was significantly smaller than the divergence of *F. prausnitzii* marker genes (**Extended Data Fig. 6b,c**). Moreover, we identified genomic islands at or near the GalNAc utilization gene cluster (**Supplementary Fig. 2**). All these findings support the potential mobilizability of the GalNAc pathway.

As recent evidence supports transmission of *F. prausnitzii* between hosts[34], we also assessed whether individuals living in the same household with individuals with the SV were also more likely to have this SV, independent of their blood group. Our analysis identified a significant effect of household SV presence, and this effect was independent of the host's genetic background (odds ratio = 2.29, $P = 3.19 \times 10^{-7}$) and additive to the genetic background effect (odds ratio = 3.27, $P < 2 \times 10^{-16}$; **Extended Data Fig. 6d**). We also compared the *F. prausnitzii* SV profiles in 119 individuals at two timepoints 4-years apart and found that the presence/absence status of the dSV region did not change for 85 (71.4%) individuals, while 23 individuals gained and 11 individuals lost the region (McNemar's one-sided test $P = 0.029$; **Extended Data Fig. 6e**).

Supplementary Figures

Supplementary Figure 1: Graphical representation of the aligned gene regions with SVs of 12 *F. prausnitzii* strains

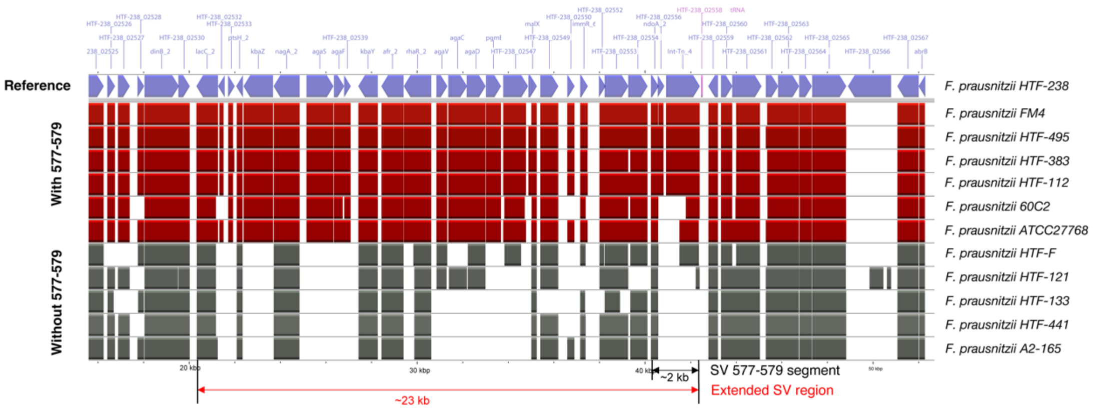

The double-headed red arrow at the bottom indicates the extension of the original SV segment 577–579 (double-headed black arrow) to a 23-kbp region. The purple arrowheads at the top depict the coding sequences of the reference genome of strain HTF-238, and the lines and protein codes above indicate gene names. Each bar below corresponds to the aligned sequence of the *F. prausnitzii* strain specified on the right. Red bars indicate strains with the GalNAc-containing region. Gray bars indicate the strains lacking these genes. The red and gray rectangles show if a gene or part of the gene is present in the genomes of strains with or without the SV respectively. Image created with CGView (https://cgview.ca).

Supplementary Figure 2: Prediction of mobilizable genomic islands in the GalNAc region of *F. prausnitzii*

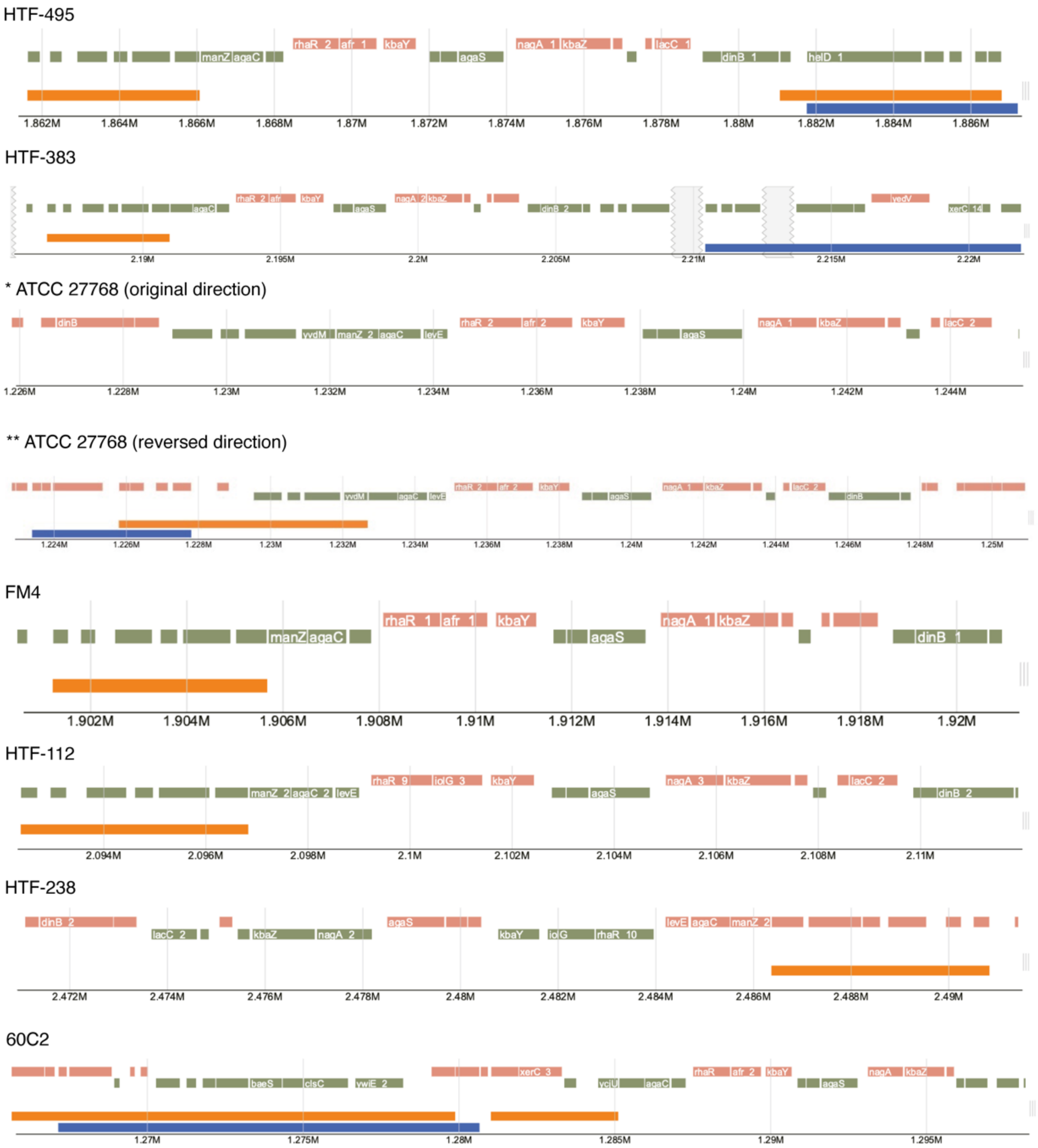

Mobilizable genomic islands (GIs) predicted for the seven GalNAc-containing *F. prausnitzii* strains. For ATCC27768, the analysis was done for both the original and reversed directions. Only the region containing GalNAc and its surrounding region are shown, along with their genomic base pair positions. The GalNAc genes are labeled and colored differently based on the transcription directions: green indicates forward transcription and red indicates backward transcription. To reduce false predictions, all genome contigs were checked by aligning them against a reference genome *F. prausnitzii* strain Indica, and the regions in HTF-383 that cannot be mapped to the reference genome are colored in gray. GI regions predicted by IslandPath-DIMOB are indicated in blue. GI regions predicted by SIGI-HMM are indicated in orange.
