# Supplementary material for: Host genetic regulation of human gut microbial structural variation
Source: Nature. 2024 Jan 3;625(7996):813–21. doi: 10.1038/s41586-023-06893-w (PMC10808065; doi:10.1038/s41586-023-06893-w)
Supplement: Supplementary file 2 — Reporting Summary [file 41586_2023_6893_MOESM2_ESM.pdf]

nature portfolio

Corresponding author(s): Jingyuan Fu

Last updated by author(s): Nov 14, 2023

Reporting Summary

Nature Portfolio wishes to improve the reproducibility of the work that we publish. This form provides structure for consistency and transparency in reporting. For further information on Nature Portfolio policies, see our Editorial Policies and the Editorial Policy Checklist.

Statistics

For all statistical analyses, confirm that the following items are present in the figure legend, table legend, main text, or Methods section.

n/a	Confirmed	
☐	☒	The exact sample size (*n*) for each experimental group/condition, given as a discrete number and unit of measurement
☐	☒	A statement on whether measurements were taken from distinct samples or whether the same sample was measured repeatedly
☐	☒	The statistical test(s) used AND whether they are one- or two-sided<br>*Only common tests should be described solely by name; describe more complex techniques in the Methods section.*
☐	☒	A description of all covariates tested
☐	☒	A description of any assumptions or corrections, such as tests of normality and adjustment for multiple comparisons
☐	☒	A full description of the statistical parameters including central tendency (e.g. means) or other basic estimates (e.g. regression coefficient) AND variation (e.g. standard deviation) or associated estimates of uncertainty (e.g. confidence intervals)
☐	☒	For null hypothesis testing, the test statistic (e.g. *F*, *t*, *r*) with confidence intervals, effect sizes, degrees of freedom and *P* value noted<br>*Give P values as exact values whenever suitable.*
☒	☐	For Bayesian analysis, information on the choice of priors and Markov chain Monte Carlo settings
☒	☐	For hierarchical and complex designs, identification of the appropriate level for tests and full reporting of outcomes
☐	☒	Estimates of effect sizes (e.g. Cohen's *d*, Pearson's *r*), indicating how they were calculated

Our web collection on statistics for biologists contains articles on many of the points above.

Software and code

Policy information about availability of computer code

Data collection	No specific software was used for data collection.
Data analysis	All data analyses were conducted using publicly available tools. For this study the following software was used: R v4.1.0, PLINK (v.alpha 2.1), KneadData v.0.7.4, Bowtie2 v.2.3.4.3, Trimmomatic v.0.39, Kraken2 v.2.1.2, Bracken v.2.6.2, MetaPhlAn v3,SGV-Finder v.1, GCTA toolbox v.1.94.1, Metal v.2020-05-05, MicrobeAnnotator v2.0.5, Bakta v1.8.1, tblastn v2.5.0+, shortBRED toolkit v.0.9.5, RAxML v8, CSI Phylogeny v1.4, IslandViewer v4. The following R packages were used: lme4qtl v.0.2.2, kinship2 v.1.9.6, vegan v.2.6-2, ape v.5.6.-2, stats v.4.3.0, ggtree v.3.2.1, gggenomes v.0.9.9.9000<br>The analysis code is available at https://github.com/GRONINGEN-MICROBIOME-CENTRE/SV_GWAS.

For manuscripts utilizing custom algorithms or software that are central to the research but not yet described in published literature, software must be made available to editors and reviewers. We strongly encourage code deposition in a community repository (e.g. GitHub). See the Nature Portfolio guidelines for submitting code & software for further information.

Data

Policy information about availability of data

All manuscripts must include a data availability statement. This statement should provide the following information, where applicable:

- Accession codes, unique identifiers, or web links for publicly available datasets
- A description of any restrictions on data availability
- For clinical datasets or third party data, please ensure that the statement adheres to our policy

The profile of SVs of all samples and the full summary statistics of genetic associations with bacterial dSVs and vSVs are available at figshare: https://doi.org/10.25452/figshare.plus.c.6877849. The assembled bacterial genomes from the growth experiment are available at NCBI with accession number PRJNA1024432.
The raw metagenomic sequencing data of all four cohorts are publicly available. Three are deposited at the European Genome–Phenome Archive: Dutch Microbiome Project (accession number EGAS00001005027), Lifelines-DEEP (accession number EGAD00001001991), and 300OB (accession number EGAD00001005083). The 500FG data is available at NCBI SRA under accession number PRJNA319574. The metagenomic data of 300TZFG is available in the NCBI BioProject under accession number PRJNA686265.
To protect participant's privacy and respect the research agreements in the informed consent, genotyping data and participant metadata are not publicly available and cannot be deposited in public repositories. The DMP and LLD data can be accessed by all bona-fide researchers with a scientific proposal by contacting the Lifelines Biobank (instructions at https://www.lifelines.nl/researcher/how-to-apply). Researchers will need to fill in an application form, which will be reviewed within 2 working weeks. If the proposed research complies with Lifelines regulations, e.g., noncommercial use and guarantee of participants' privacy, researchers will then receive a financial offer and a data and material transfer agreement to sign. In general, data will be released within 2 weeks after signing the offer and data and material transfer agreement. The data will be released in a remote system (the Lifelines workspace) running on a high-performance computer cluster to ensure data quality and security. As Lifelines is a non-profit organization dependent on (governmental) subsidies, a fee is required to cover the costs of controlled data access and supporting infrastructure. The fee for data access on the HPC is €3,500 for 1 year and the fee for the Lifelines Workspace environment is €4,500 for 1 year, or less for shorter periods of time. There are no restrictions on the downstream re-use of aggregated, non-identifiable results (as approved by Lifelines), nor are there authorship requirements, but Lifelines does request that it is acknowledged in publications using these data. The data access policy, data access fees and an example Data and Material Transfer Agreement (which includes details on how to acknowledge the use of Lifelines data in publications) are described in detail at https://www.lifelines.nl/researcher/how-to-apply. Note that data access for replication can be arranged via Lifelines. Lifelines will not charge an access fee for controlled access to the full dataset used in the manuscript (including phenotype and sequencing data), for the specific purpose of replication of the results presented in this Article or for further assessment by the reviewers, for a period of three months. Researchers interested in such a replication study or review assessment can contact Lifelines at research@lifelines.nl.
The genotype and metadata of 500FG, 300OB, and 300TZFG cohorts can be requested via the Human Functional Genomics Data Access Committee (Martin Jaeger, e-mail: Martin.Jaeger@radboudumc.nl)]. There are no conditions associated with its use, with the exception of those associated with data that may lead to compromising patient confidentiality, such as raw genomics data. The data are freely available, and no agreement or costs are required. The applicants would receive a response within 4 weeks from application.
Gut microbial SV calling was conducted based on reference microbial genomes from the proGenomes database (http://progenomes1.embl.de/). ShortBRED analysis was performed based on the UniRef90 database (https://ftp.uniprot.org/pub/databases/uniprot/uniref/uniref90/).

Human research participants

Policy information about studies involving human research participants and Sex and Gender in Research.

Reporting on sex and gender	We added sex as covariate in association analyses as the primary goal of the project was to identify associations between host and microbial genetic variants irrespective of sex.
Population characteristics	Data from five population-based cohorts were used in this study.<br>DMP cohort is a prospective cohort from the north of the Netherlands that consists of 8,719 individuals. 57.4% of participants are female, the mean age (SD) of participants is 48.42 (14.79) years, mean BMI is 25.56 (4.40).<br>LLD cohort is a prospective cohort from the north of the Netherlands that consists of 1,135 individuals. 58.20% of participants are female, the mean age (SD) of participants is 45.04 (13.60) years and their mean BMI is 25.26 (4.18).<br>The 500FG cohort consists of 534 healthy adult volunteers from the Netherlands. 56.50% of participants are female, the mean age of participants is 27.43 (12.35) years and their mean BMI is 22.70 (2.72).<br>The 300OB is a part of the Functional Genomics project and consists of 302 individuals from the Netherlands with a BMI >27. 44.30% of participants are female, the mean age of participants is 67.07 (5.39) years and their mean BMI is 30.73 (3.48).<br>The 300TZFG cohort consists of 323 individuals from both rural (N = 70, median age 39.6) and urban (N = 253, median age 27.6) areas of Tanzania. A total of 279 individuals with both genotype and metagenomics data are included in the study.
Recruitment	DMP and LLD are subsets of the Lifelines cohort, which has been recruited in three stages: recruitment of an index population via general practitioners, subsequent inclusion of their family members, and online self-registration. This cohort is considered representative of adult population of the North of the Netherlands. 500FG and 300OB cohorts are part of the Functional Genomics project. The inclusion of the volunteers took place between 8/2013 until 12/2014 in the Radboud University Medical Center, the Netherlands. 500FG is a population-based cohort, while around half of 300OB participants are clinically diagnosed with metabolic syndrome.<br>The participants of 300TZFG were recruited from the Kilimanjaro region of the Northern Tanzania between March and December 2017, at the Kilimanjaro Christian Medical Center and Lucy Lameck Research Center, in Moshi municipal. Exclusion criteria were pregnancy, a known acute or chronic disease, use of antibiotics or anti-malarials in the previous three months, or receiving treatment for tuberculosis infection in the past year. The information on the study was given through leaflets or announced during the mass gathering. All volunteers were interviewed by a member of the study team using a guided pre-screening questionnaire prior to being invited to the study center.
Ethics oversight	The Lifelines study was approved by the ethics committee of the University Medical Center Groningen (METc2007/152). All

Ethics oversight	participants signed an informed consent form prior to enrollment. Additional written consents were signed by the DMP participants or legal representatives for children aged under 18 years. The Lifelines-DEEP study was approved by the Institutional Ethics Review Board of the University Medical Center Groningen (ref. M12.113965), the Netherlands. The 300-Obesity study was approved by the IRB CMO Regio Arnhem-Nijmegen (nr. 46846.091.13). The 500FG study was approved by the Ethical Committee of Radboud University Nijmegen (NL42561.091.12, 2012/550). The inclusion of volunteers and experiments were conducted according to the principles expressed in the Declaration of Helsinki. All volunteers gave written informed consent before any material was taken.<br>The 300TZFG study was approved by the Ethical Committees of the Kilimanjaro Christian Medical University College (CRERC) (no. 936) and the National Institute for Medical Research (NIMR/HQ/R.8a/Vol. IX/2290) in Tanzania.

Note that full information on the approval of the study protocol must also be provided in the manuscript.

Field-specific reporting

Please select the one below that is the best fit for your research. If you are not sure, read the appropriate sections before making your selection.

☒ Life sciences ☐ Behavioural & social sciences ☐ Ecological, evolutionary & environmental sciences

For a reference copy of the document with all sections, see nature.com/documents/nr-reporting-summary-flat.pdf

Life sciences study design

All studies must disclose on these points even when the disclosure is negative.

Sample size	In order to ensure the analysis power, the study includes as much as subjects as possible from the five cohorts. Thus no sample size calculation was performed. For each cohort we used samples that had both metagenomic and genotype data available: DMP (N = 7,372), LLD (N = 981), 500FG (N = 396), 300OB (N = 266), and 300TZFG (N = 279).
Data exclusions	We excluded samples with < 5% of structural variations called.
Replication	We used all available samples of four independent Dutch cohorts for meta-analysis. To ensure the replication of the identified associations, we required that the associations were not only significant at Bonferroni-corrected P <0.05 in meta-analysis but also nominally significant (p < 0.05) in at least two cohorts with consistent effect direction. The 300TZFG was used as an extra independent replication cohort in non-European population, with a nominally significant threshold (p < 0.05).
Randomization	This is human cohort-based analysis. The sample collection and sequencing were performed in a random order. No extra randomization was done for this study.
Blinding	This study is a human cohort based, observational study. Thus no blinding was performed.

Reporting for specific materials, systems and methods

We require information from authors about some types of materials, experimental systems and methods used in many studies. Here, indicate whether each material, system or method listed is relevant to your study. If you are not sure if a list item applies to your research, read the appropriate section before selecting a response.

Materials & experimental systems

n/a	Involved in the study	
☒	☐	Antibodies
☒	☐	Eukaryotic cell lines
☒	☐	Palaeontology and archaeology
☒	☐	Animals and other organisms
☒	☐	Clinical data
☒	☐	Dual use research of concern

Methods

n/a	Involved in the study	
☒	☐	ChIP-seq
☒	☐	Flow cytometry
☒	☐	MRI-based neuroimaging
